# False-positive cervical lymph nodes on FAPI PET/CT after neoadjuvant chemotherapy for breast cancer: a case report

**DOI:** 10.3389/fonc.2026.1938265

**Published:** 2026-09-22

**Authors:** Ying Liu, Xiaowei Ma, Jiaying Yang, Jiqin Yang, Qian Zhao

**Affiliations:** Department of Nuclear Medicine, General Hospital of Ningxia Medical University, Yinchuan, China

**Keywords:** breast cancer, false positive, FAPI PET/CT, inflammatory lymphadenopathy, neoadjuvant chemotherapy

## Abstract

A 49-year-old woman with cT3N1M0 stage IIIA HER2-positive invasive breast cancer is described in this retrospective case report. After six cycles of TCHP neoadjuvant chemotherapy, breast MRI confirmed that the primary lesion and axillary lymph nodes achieved radiological complete response. However, newly enlarged lymph nodes in levels II and III of the left neck were detected by neck ultrasound and contrast-enhanced CT. Subsequent 18F-FAPI-04 PET/CT revealed significantly elevated tracer uptake in these cervical lymph nodes, with a maximum standardized uptake value (SUVmax) of 18.2, which was highly suspicious for metastasis. Ultrasound-guided core needle biopsy and postoperative histopathological examination finally confirmed that these cervical lymph nodes were benign reactive lymphoid hyperplasia with focal necrosis and inflammatory cell infiltration, and the patient achieved pathological complete response in both breast and axillary regions without any lymph node metastasis. This case reveals that despite the high sensitivity of FAPI PET/CT in detecting malignant tumor lesions, benign post-treatment inflammatory processes can induce significant FAP upregulation, leading to typical false-positive results. Clinical interpretation of FAPI PET/CT findings must be closely integrated with the patient’s treatment history and multimodality examinations, and histopathology should remain the gold standard for definitive lesion characterization to avoid unnecessary misdiagnosis and overtreatment.

## Introduction

Breast cancer remains the most commonly diagnosed malignancy and the leading cause of cancer-related death among women worldwide (1). Neoadjuvant chemotherapy (NAC) has become a standard treatment strategy for locally advanced breast cancer (2). Pathological complete response (pCR) after NAC is recognized as a robust prognostic marker (3, 4).

Positron emission tomography/computed tomography (PET/CT) with 18F-fluorodeoxyglucose (FDG) has been widely used for staging, response assessment, and surveillance in breast cancer patients. However, FDG PET/CT has inherent limitations including physiological uptake in brain and urinary tract, inflammatory false positives, and reduced sensitivity in low-metabolic tumor subtypes (5).

Fibroblast activation protein inhibitor (FAPI) PET/CT has emerged as a promising novel molecular imaging modality targeting cancer-associated fibroblasts (CAFs). FAP is a type II transmembrane serine protease overexpressed in the stroma of approximately 90% of epithelial malignancies but shows minimal expression in normal adult tissues (6). Preclinical and early clinical studies have demonstrated that FAPI PET/CT exhibits high tumor-to-background ratio (7). In breast cancer specifically, FAPI PET/CT has shown potential utility in primary tumor detection, lymph node staging, and treatment response monitoring (8).

Despite its promising sensitivity, emerging evidence indicates that FAPI uptake is not entirely tumor-specific. Activated fibroblasts are also present in benign conditions including inflammation, tissue repair, fibrosis, and granulomatous diseases, which can lead to false-positive interpretations (9). This is particularly relevant in the post-treatment setting where therapy-induced inflammatory changes may confound image interpretation.

Herein, we present a clinically instructive case of a HER2-positive breast cancer patient who achieved pCR after NAC but developed newly enlarged cervical lymph nodes with intense FAPI uptake that were ultimately confirmed as inflammatory hyperplasia rather than metastasis. This case highlights important diagnostic pitfalls of FAPI PET/CT interpretation in the post-NAC setting and underscores the critical importance of histopathological correlation.

## Case report

A 49-year-old woman presented to our hospital in early 2025 with a palpable left breast mass of one month’s duration. After a series of examinations, she was diagnosed with: 1) Left-sided invasive breast carcinoma of no special type (cT3N1M0, stage IIIA, HER2-positive) with ipsilateral axillary lymph node metastasis. 2) Right thyroid nodule (TI-RADS 4a).

Prior to NAC, ultrasound-guided core needle biopsy of the left breast mass and a left axillary lymph node was performed to establish the diagnosis. Histopathology showed: (1) Left breast: a small amount of fibrotic stromal tissue containing invasive carcinoma of no special type, grade III (Nottingham histological score 3 + 3 + 2 = 8); (2) Left axillary lymph node: carcinoma arranged in a glandular/tubular pattern within cord-like fibrous and lymphoid tissue, consistent with metastatic breast carcinoma on immunohistochemistry. Immunohistochemistry of the primary breast carcinoma (slide 2) demonstrated: P63(-), Calponin(-), ER(<1%), PR(<1%), AR(90%, 3+), C-erbB-2(90%, 3+), CK5/6(-), Ki67 (index ~30%), P120(membranous+), E-cadherin(membranous+), GST-pi(-), TOP-II(20%, +), TRPS1(+), GATA-3(+), CD34(-), D2-40(-), S100(-), MUC1(+). The metastatic axillary carcinoma (slide 6) showed: ER(30%, 1+), PR(<1%), C-erbB-2(90%, 3+), Ki67 (index ~20%), CK7(+), GATA-3(+), TRPS1(+), CK5/6(-).

The patient successfully completed six cycles of TCHP (docetaxel, carboplatin, trastuzumab, and pertuzumab) NAC. Preoperative breast MRI indicated a complete response (CR) of the left breast lesion and left axillary metastatic lymph nodes based on MRI response evaluation criteria following neoadjuvant chemotherapy (NAC). However, both neck ultrasonography and enhanced neck CT identified multiple newly enlarged lymph nodes in the left cervical levels II and III, some with necrotic changes, raising suspicion for metastasis. A 0.3 cm x 0.4 cm low-density nodule in the right thyroid lobe (TI-RADS 4a) showed no change in size compared with previous ultrasound (**Figure 1**).

**Figure 1:**
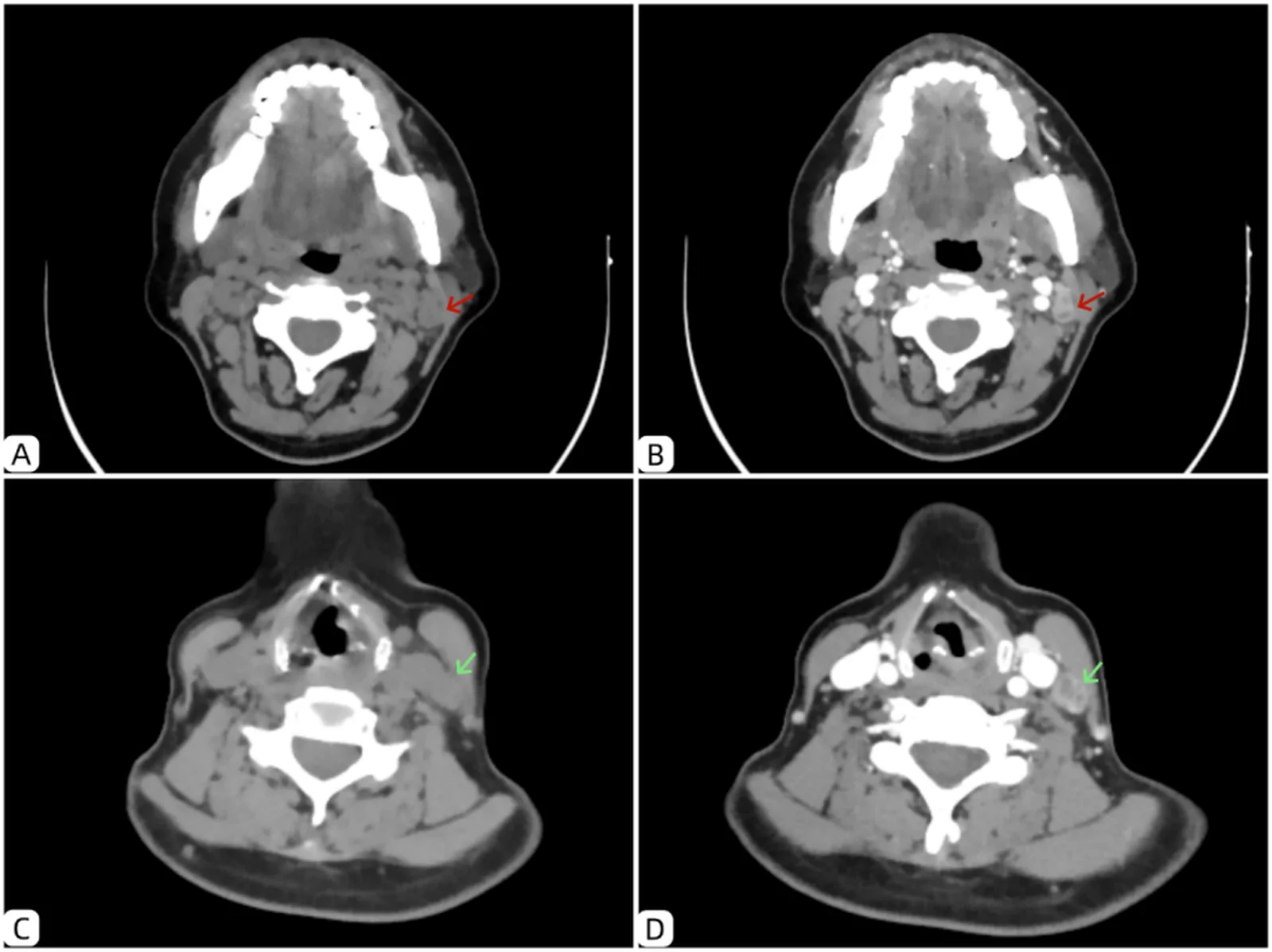
Plain and contrast-enhanced cervical CT images. **(A, C)** non-contrast CT scans, with arrows marking the enlarged lymph nodes in level II and level III of the left neck. **(B, D)** contrast-enhanced cervical CT images, with arrows pointing to the unevenly enhanced enlarged left cervical lymph nodes with internal necrotic foci.

We scheduled 18F-FAPI-04 PET/CT scanning roughly three weeks after the patient finished six cycles of neoadjuvant chemotherapy to clarify whether enlarged neck nodes represented metastatic lesions. The patient maintained fasting for more than six hours prior to imaging. All scans were acquired on a GE Max+ hybrid PET/CT scanner manufactured by GE Healthcare. A dose of 5.65 mCi 18F-FAPI-04 was administered intravenously, adjusted to body weight; imaging was initiated 50 minutes after injection. CT acquisition parameters were: tube voltage 120 kV and automatic tube current modulation with a slice thickness of 3 mm. PET emission data were acquired in 3D mode. Images were reconstructed using an ordered subset expectation maximization (OSEM) algorithm with SUV normalization to body weight. Two experienced nuclear medicine physicians independently reviewed all images, with discrepancies resolved by consensus.

The scan report (**Figures 2**, **3**) indicated enlarged left cervical lymph nodes in levels II and III with increased FAPI uptake, the largest in the left cervical level II measuring about 1.2 cm x 1.0 cm with SUVmax reaching 18.2, suspicious for metastasis. The low-density focus in the inferior pole of the right thyroid lobe showed mild FAP expression, diameter ~0.4 cm, SUVmax 3.4, suspicious for malignancy.

**Figure 2:**
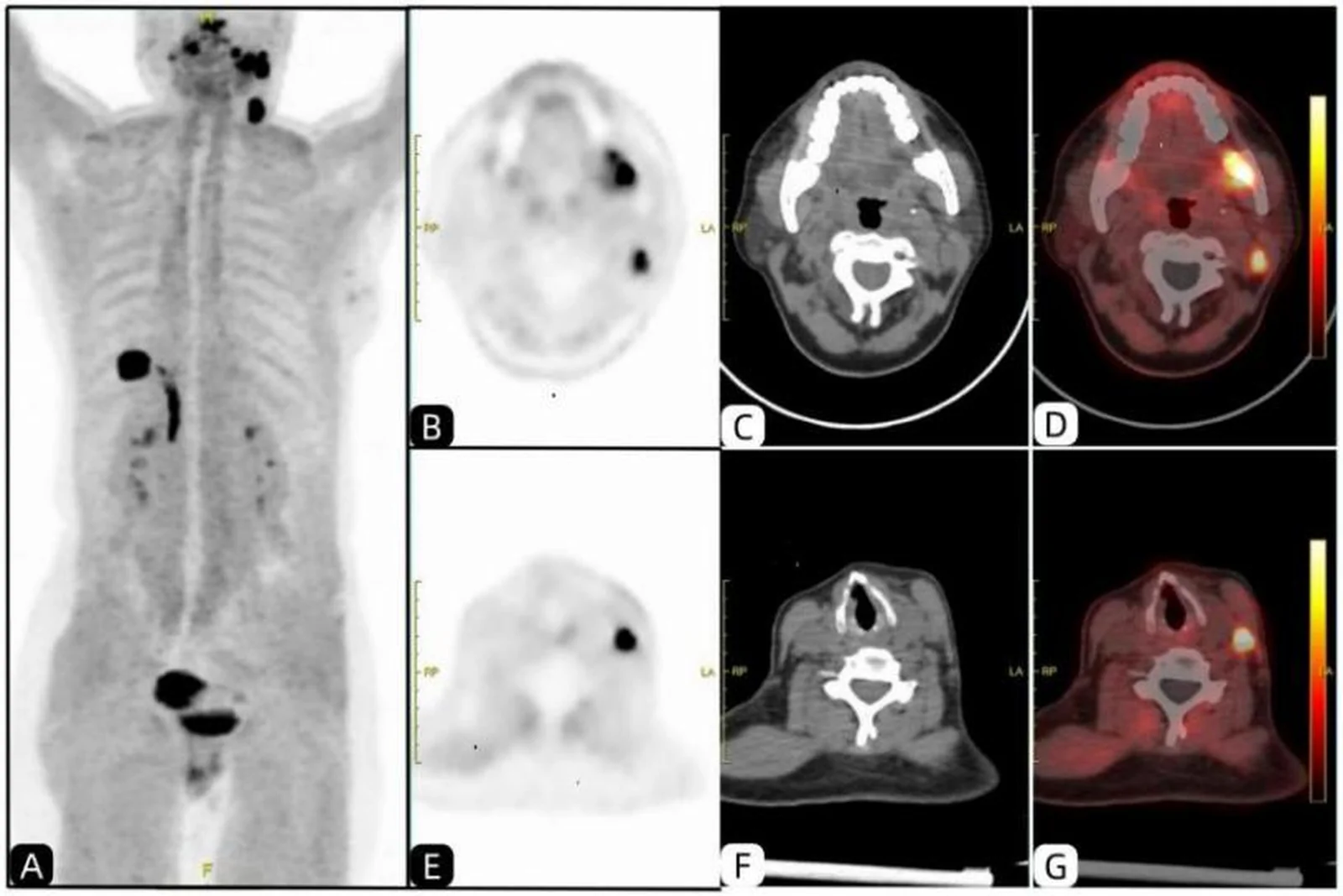
18F-FAPI PET/CT images. **(A)** Maximum intensity projection (MIP) image, showing multiple nodular radiotracer accumulation foci in the left cervical region. **(B, E)** Transaxial PET images of the neck. **(C, F)** CT images at the identical anatomical levels. **(D, G)** Fused PET/CT images of the neck. The images demonstrated multiple lymph nodes with elevated radiotracer uptake in levels II and III of the left neck. The largest one was located in level II of the left neck, measuring approximately 1.2 cm x 1.0 cm, with an SUVmax of 18.2.

**Figure 3:**
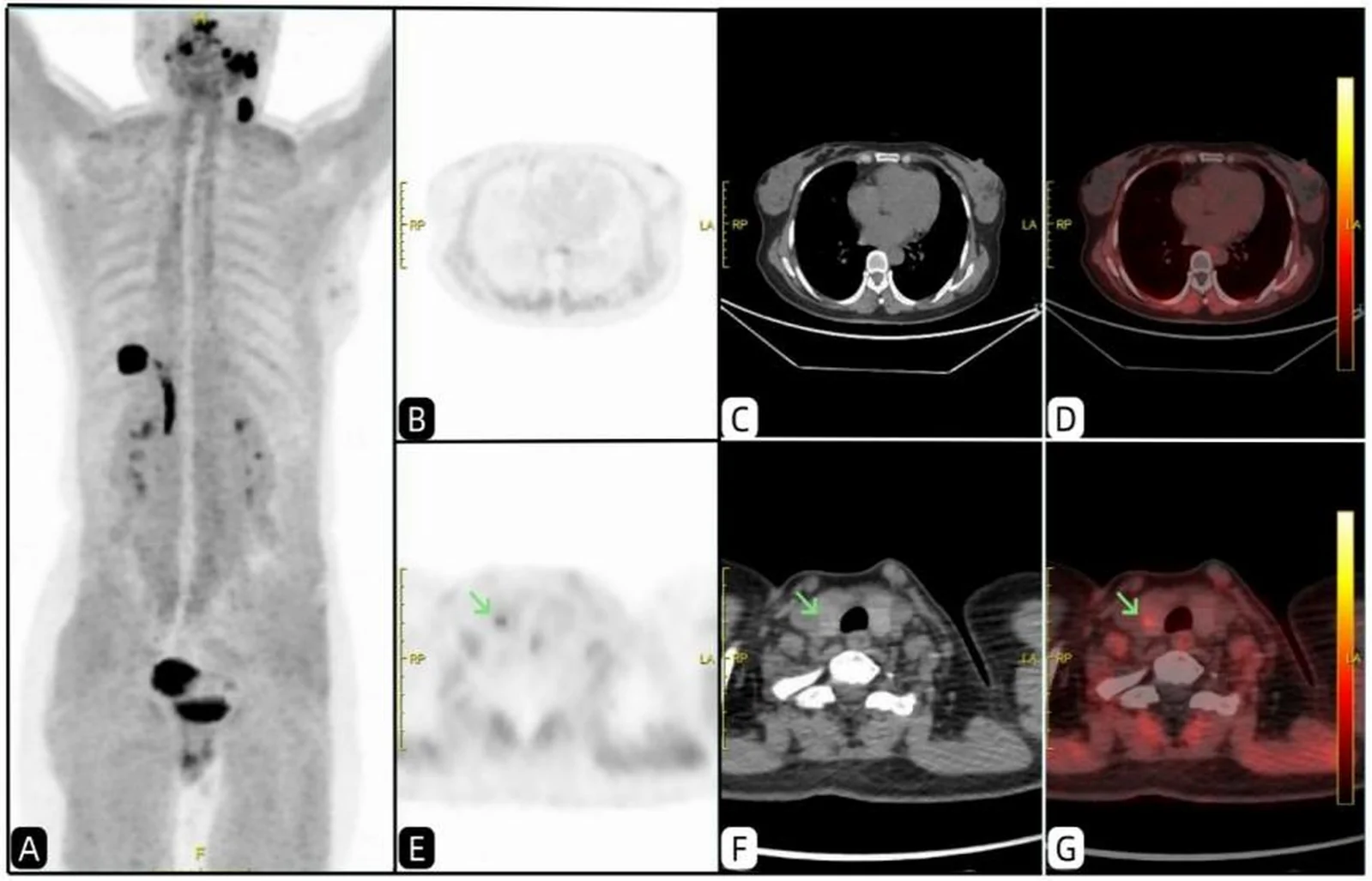
18F-FAPI PET/CT images. **(A)** MIP image. **(B–D)** Transaxial thoracic images showing post-chemotherapy changes in the left breast region, with no neoplastic lesions or abnormal radiotracer uptake observed. **(E–G)** Similarly, the transaxial cervical images revealing an abnormal low-density nodule with mild FAPI uptake in the right thyroid lobe (green arrow, SUVmax 3.4).

Core needle biopsy of a left cervical lymph node subsequently returned a histopathological diagnosis of reactive lymphoid hyperplasia (**Figure 4**). Pathological observation of puncture tissue showed cord-shaped lymphoid structures, accompanied by local necrotic foci. Numerous inflammatory cells infiltrated the fibrous stroma and its surrounding regions, yet no malignant tumor cells could be detected in any of the examined fields. We did not carry out additional special histochemical or immunohistochemical staining on this biopsy sample. It is worth noting that the postoperative excised neck lymph nodes shared identical pathological features with the puncture specimen, all presenting reactive lymphoid hyperplasia without metastatic carcinoma. This consistency verified that the needle biopsy obtained representative tissue for diagnosis. In August 2025, the patient underwent “left modified radical mastectomy, right thyroid lobectomy with isthmusectomy, and left cervical lymph node dissection (levels II, III, and IV).” Our multidisciplinary team reached a consensus to carry out combined surgical treatment after full discussion, based on three key clinical factors. First, the patient had a pre-existing TI-RADS 4a nodule in the right thyroid lobe, which met the surgical indication for resection. Second, the FAPI PET/CT scan showed extremely high tracer uptake (SUVmax=18.2) in cervical nodes, which sharply conflicted with the benign result from needle biopsy; we worried that limited puncture tissue might miss focal metastatic lesions due to sampling bias. Third, thorough surgical resection could obtain complete lymph node tissue for definitive pathological diagnosis, which helped us rule out hidden metastasis and formulate accurate postoperative adjuvant therapy schemes. The post-neoadjuvant modified radical mastectomy specimen showed: breast parenchyma with scattered foamy cells and histiocytic reaction, focal calcification, and inflammatory infiltrate representing post-chemotherapy changes; all surgical margins (peripheral and deep) were free of carcinoma; the nipple and skin were uninvolved; and no abnormality was identified in the surrounding breast tissue. The tumor bed measured a maximum of 14 mm x 14 mm, with approximately 0% viable tumor cells and 0% *in situ* carcinoma. The residual cancer burden (RCB) score was 0 (pathological complete response). Of the left axillary lymph nodes examined, 0/14 were positive for metastasis. The final pathology also revealed: 2) Right thyroid papillary carcinoma, follicular variant, tumor diameter 0.3 cm, staged as AJCC pT1aN0. 3) No metastatic carcinoma found in the dissected cervical lymph nodes (right central neck 0/6; left neck level III 0/13, left neck level IV 0/2; left neck level II nodes also negative).

**Figure 4:**
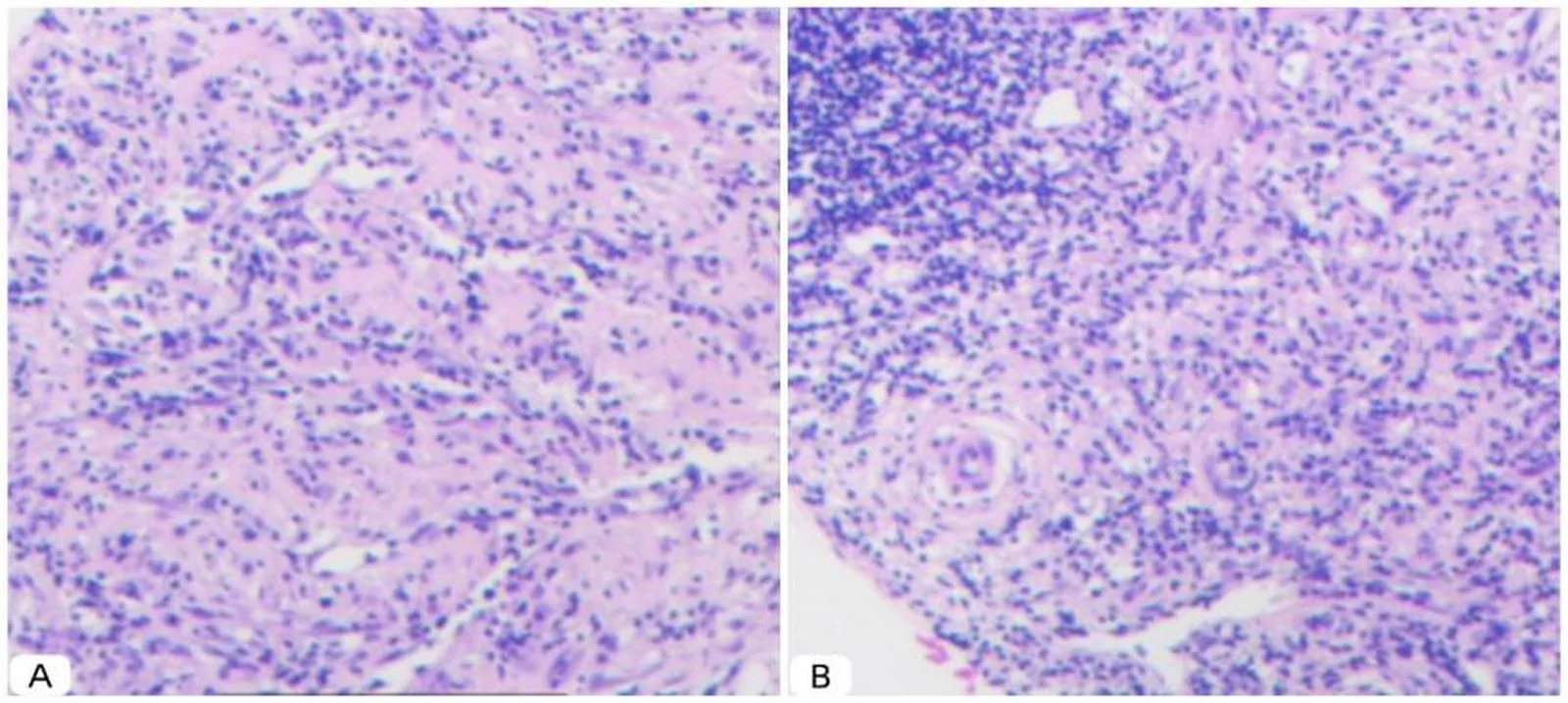
Hematoxylin-eosin (HE) stained pathological sections of cervical lymph node puncture specimens (original magnification x100 and x400). Panels **(A, B)** show typical reactive lymphoid hyperplasia: the basic nodal structure remains intact, accompanied by follicular hyperplasia and expanded paracortical zones, with inflammatory cord-like lymphoid tissue and focal necrosis in partial regions. A large number of inflammatory cell infiltrations could be observed in and around the fibrous stroma, and no tumor cells were detected.

Follow-up until February 2026 (approximately 6 months post-surgery), with three-monthly comprehensive re-examinations, showed no signs of tumor recurrence or metastasis in the left breast, and no abnormal cervical lymph nodes (**Table 1**). Each follow-up visit comprised physical checkups, breast and chest CT scans, neck ultrasonography, serum tumor marker detection (CEA, CA15-3, CA125), and thyroid function tests. All test results stayed within the normal reference range during all follow-up visits. The patient is receiving standardized postoperative treatment, combining aromatase inhibitor endocrine therapy with one year of adjuvant anti-HER2 targeted therapy (trastuzumab) following clinical guidelines. Up to the latest follow-up, no adverse reactions linked to surgery or postoperative medication have occurred. The patient’s physical condition remained stable (ECOG score 0), and she continues regular long-term surveillance as recommended by NCCN breast cancer guidelines.

**Table 1:** Chronological timeline of diagnosis and treatment of the patient.

Date	Event	Key findings
Early 2025	Initial presentation	Left breast mass; cT3N1M0 HER2+ invasive breast cancer; right thyroid nodule TI-RADS 4a
Early 2025	Baseline workup & pre-NAC biopsy	Core biopsy: IDC grade III (Nottingham 3 + 3 + 2 = 8); axillary node metastases confirmed by IHC; full IHC profile obtained
2025 (6 cycles)	NAC	TcbHP regimen; tolerated well
Late Jul 2025	Post-NAC imaging	Breast MRI: CR; Neck US/CT: new enlarged left cervical LNs
~3 weeks post-NAC (Aug 2025)	FAPI PET/CT	Left cervical level II LN SUVmax 18.2; thyroid SUVmax 3.4
Aug 2025	Core needle biopsy	Left cervical LN: reactive lymphoid hyperplasia (no special staining)
Aug 2025	MDT discussion	Decision for comprehensive surgery
Aug 2025	Surgery	Left mastectomy + right thyroid lobectomy with isthmusectomy + left cervical dissection (levels II-IV)
Aug 2025	Final pathology	Breast/axilla: pCR, RCB 0; axilla 0/14; Thyroid: follicular variant papillary Ca, pT1aN0; cervical LNs all benign
Aug 2025-Feb 2026	Adjuvant therapy	Endocrine therapy + trastuzumab; surveillance
Feb 2026	Latest follow-up	No recurrence; ECOG 0; continuing adjuvant therapy

## Chronological timeline of diagnosis and treatment

## Discussion and conclusion

pCR serves as a critical positive prognostic indicator for outcome evaluation after NAC in breast cancer, and patients who achieve pCR demonstrate a remarkably reduced long-term risk of recurrence and metastasis (3, 10). Although isolated case reports have documented regional lymph node recurrence following pCR in breast cancer patients treated with NAC, distant cervical lymph node metastasis remains an extremely rare clinical scenario (11, 12). In this case, both the primary breast lesion and axillary metastatic lymph nodes of the patient achieved pathological complete response (pCR) after NAC treatment. From the perspective of disease progression pattern, the probability of newly developed metastatic lymph nodes in the ipsilateral neck was extremely low. However, the patient also had a TI-RADS 4a micro-nodule in the right thyroid lobe. From the perspective of tumor metastatic pathways, the probability of metastasis to contralateral lymph nodes was extremely low (13), which did not conform to the typical metastatic pattern and presented a clear contradiction with the “metastasis” signs indicated by imaging in this case. How to effectively distinguish inflammatory hyperplasia from tumor metastasis and avoid missed diagnosis and overtreatment is the core priority of diagnosis and management in this case.

FAPI PET/CT targets Fibroblast Activation Protein (FAP) in the tumor microenvironment (14). FAP is highly expressed in the stroma of approximately 90% of malignant solid tumors but shows low or absent expression in normal tissues (6). This characteristic confers two primary advantages. Firstly, it demonstrates higher sensitivity than FDG PET/CT for detecting various subtypes of breast cancer and small metastatic lesions, exhibiting superior accuracy in certain contexts (15). Secondly, it effectively circumvents the issue of missed lesions associated with suppressed tumor cell glucose metabolism post-chemotherapy that can affect FDG PET/CT (5). Additionally, its clinical utility is enhanced by features such as no need for special patient preparation and generally low background signal, which help ensure accurate post-NAC tumor staging.

Clinicians need to recognize a key limitation of FAPI PET/CT: this imaging tool delivers excellent sensitivity for malignant lesions, yet it lacks ideal specificity. Fibroblasts can become activated under multiple benign pathological states, such as local inflammation, tissue repair after injury, fibrotic changes and granulomatous lesions, which may produce positive tracer signals (16). We cannot simply conclude that FAPI PET/CT is superior to FDG PET/CT under all clinical scenarios. Imaging selection must be tailored according to each patient’s unique disease background and clinical demands. Leveraging these advantages, the patient underwent FAPI PET/CT imaging after providing written informed consent. It revealed high uptake (SUVmax 18.2) in the multiple enlarged left cervical lymph nodes (levels II and III) and mild FAP expression in the right thyroid lobe. No other foci of high FAP uptake were detected elsewhere in the body. However, the final pathology for the left cervical lymph nodes showed inflammatory hyperplasia, not metastasis. Existing studies have confirmed that multiple benign pathological processes, including infection, granulomatous lesions, local tissue repair and inflammatory response after anti-tumor therapy, could induce the upregulation of local FAP expression, resulting in false-positive imaging on FAPI PET/CT (14, 16). This limitation has also been noted in other solid tumors. For instance, post-therapy sarcoid-like granulomatous inflammation and treatment-related tissue repair reactions have been reported to cause high FAPI uptake mimicking metastatic lesions, confounding clinical judgment (17, 18). Similar pseudoprogression phenomena have been reported in neoadjuvant immunochemotherapy for non-small cell lung cancer (NSCLC) (19), while non-metastatic lymph nodes with high FAPI uptake after NAC for breast cancer are rarely reported in the existing literature.

Based on the analysis of the diagnosis and treatment course of this case, after receiving high-intensity NAC, one possible mechanism is that the patient’s immune system activation and/or potential subclinical infection induced inflammatory hyperplasia of cervical lymph nodes and led to high FAP expression, which caused FAPI PET/CT to misclassify the lesion as metastatic. We acknowledge that this proposed mechanism remains speculative in the absence of specific laboratory evidence (such as inflammatory markers, microbiological cultures, or immunophenotyping) to definitively support immune activation or subclinical infection as the causative factor. Alternative explanations include treatment-related tissue remodeling and reactive lymphoid hyperplasia (14, 16) independent of identifiable infectious triggers. This is a typical clinical manifestation of the insufficient specificity of this technology.

Overall, FAPI PET/CT offers the advantages of one-stop whole-body imaging and high detection rate for tiny lesions (6). It can provide critical imaging support for whole-body lesion screening and differential diagnosis of multiple lesions in tumor patients, effectively optimizing the tumor staging evaluation system. However, this technique has an inherent defect of limited specificity, as benign inflammatory lesions can trigger false-positive results and easily interfere with clinical diagnosis and treatment decision-making. Therefore, in clinical practice, its diagnostic and therapeutic value should be assessed critically, and it is not advisable to formulate treatment plans solely relying on molecular imaging findings.

When encountering newly enlarged cervical lymph nodes with intense FAPI uptake in breast cancer patients after neoadjuvant chemotherapy, clinicians need to consider the differential diagnoses systematically. Reactive lymphadenopathy ranks first among benign causes, which usually arises from local irritation or systemic inflammatory stimulation. Systemic infectious diseases are another possibility. Bacterial, viral or fungal pathogens may trigger lymphadenitis, with or without pus formation in lymph node tissue. Granulomatous disorders such as sarcoidosis (18) and tuberculosis also lead to lymph node enlargement via granuloma formation inside nodal tissue. We cannot ignore inflammatory reactions induced by chemotherapy itself: NAC may activate systemic immune responses, remodel interstitial tissue, and even trigger sarcoid-like granulomatous lesions. Though the patient obtained pathological complete response and the location of enlarged neck nodes did not match typical breast cancer metastatic routes, we still could not rule out breast cancer metastasis without pathological evidence. The patient carried a suspicious thyroid nodule simultaneously, so metastatic spread from papillary thyroid carcinoma to cervical lymph nodes also needed exclusion, as thyroid malignancies follow fixed lymphatic drainage patterns (13). Besides the above lesions, primary or secondary lymphoma should also be considered for patients presenting with painless swollen neck lymph nodes. Only tissue pathological testing can differentiate all these diseases and reach a definitive diagnosis. In conclusion, for atypical new imaging lesions that emerge during the follow-up period after NAC for tumors, clinicians should adopt a prudent diagnostic and therapeutic mindset. They should complete preliminary screening by combining highly sensitive molecular imaging techniques such as FAPI PET/CT with multimodality imaging examinations, and ultimately take histopathological biopsy as the gold standard for determining the nature of lesions. Meanwhile, attention should be paid to the evaluation and standardized management of the patient’s concurrent second primary tumor, to minimize the risks of misdiagnosis, missed diagnosis and overtreatment.

This study was a retrospective single-case report with a limited sample size, so there were certain limitations in the extrapolation of its research conclusions. At present, unified standards have not yet been formed for the optimal application scenarios and precise diagnostic thresholds of FAPI PET/CT (17) in differentiating inflammatory hyperplasia from tumor metastasis after breast cancer treatment, which still need further exploration and verification through large-sample, prospective clinical studies. In the diagnosis and treatment system of precision oncology, the organic integration of functional molecular imaging and histopathology, as well as the application of multidisciplinary team (MDT) diagnosis and treatment mode, is key to optimizing individualized diagnosis and treatment regimens, avoiding clinical pitfalls and improving patient prognosis.

## Data Availability

The raw data supporting the conclusions of this article will be made available by the authors, without undue reservation.
